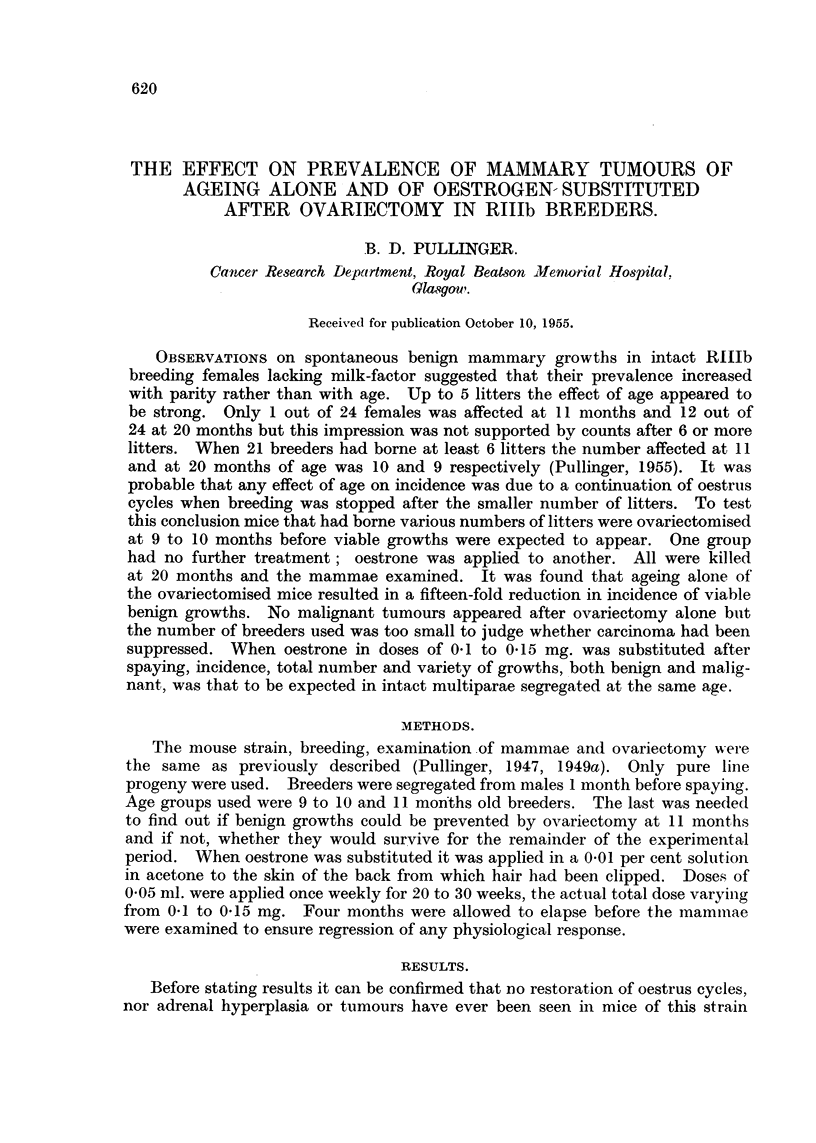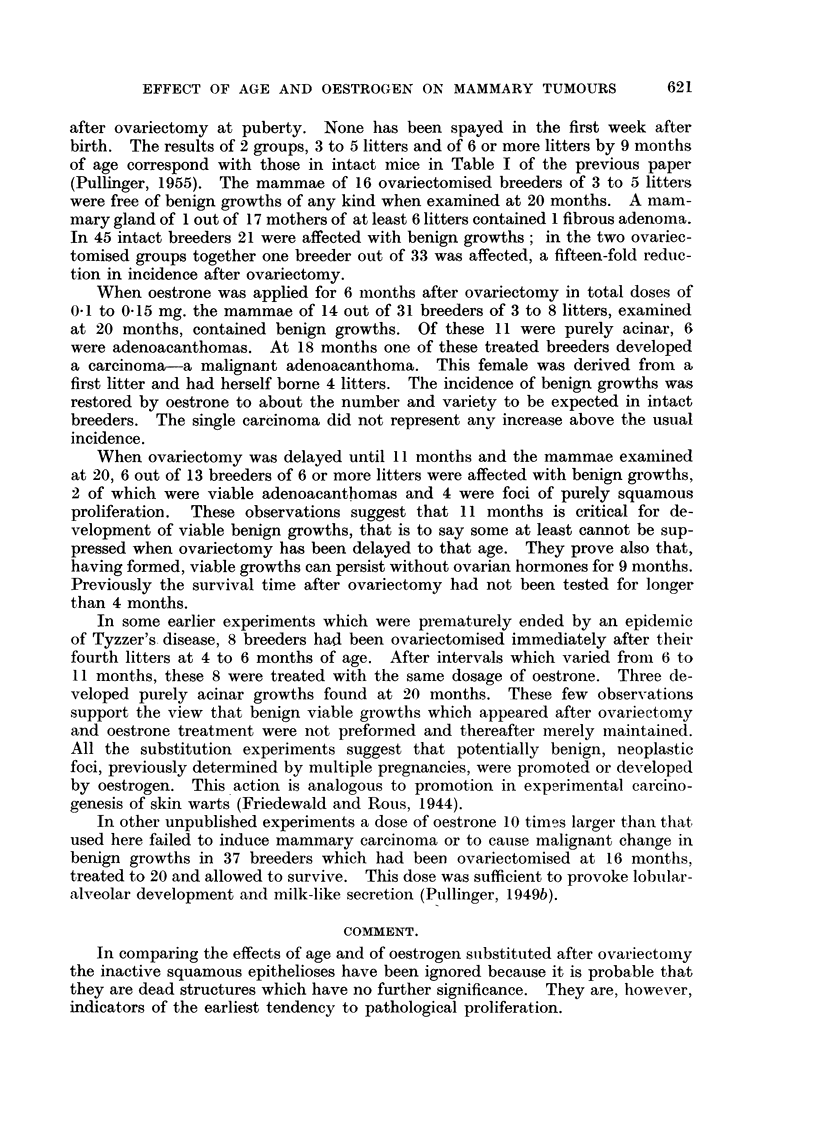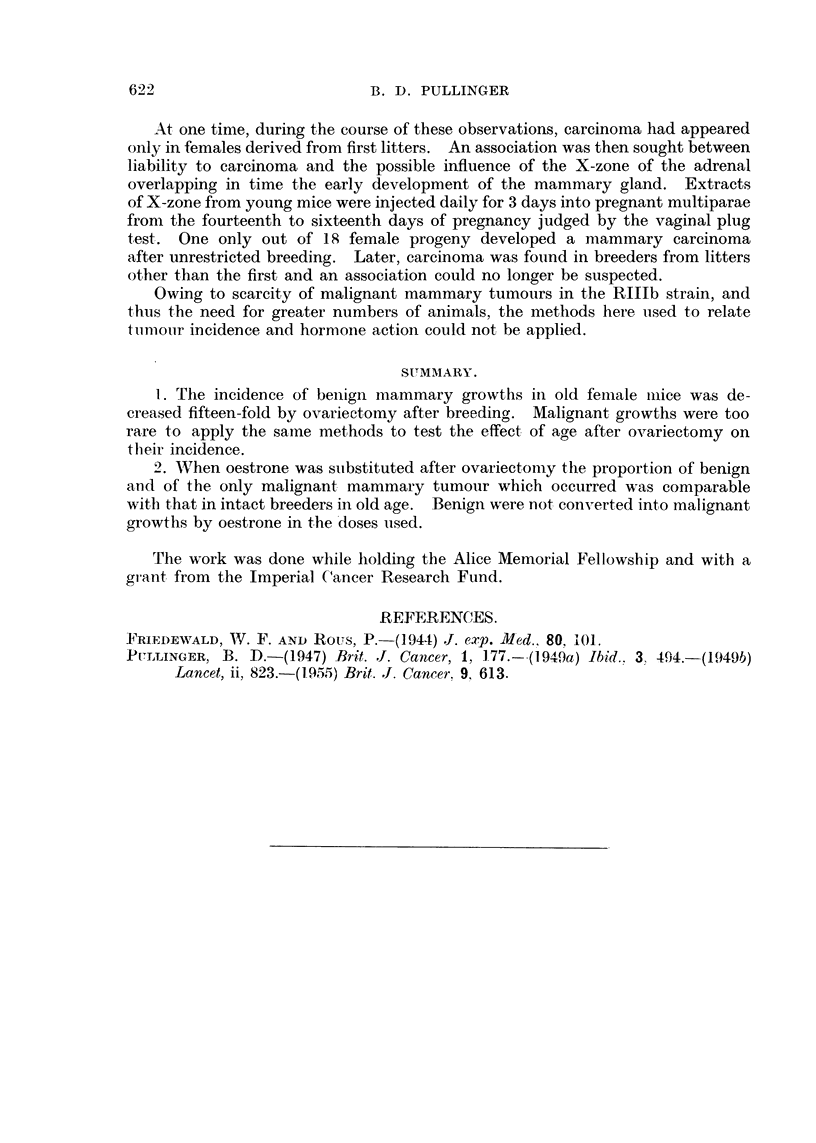# The Effect on Prevalence of Mammary Tumours of Ageing Alone and of Oestrogen Substituted After Ovariectomy in RIIIb Breeders

**DOI:** 10.1038/bjc.1955.65

**Published:** 1955-12

**Authors:** B. D. Pullinger


### THE EFFECT ON PREVALENCE OF MAMMARY TUMOURS OF AGEING ALONE AND OF OESTROGEN-SUBSTITUTED AFTER OVARIECTOMY IN RIIIb BREEDERS.

B. D. PULLINGER.

*Cancer Research Department, Royal Beatson Memorial Hospital, Glasgow.*

Received for publication October 10, 1955.

OBSERVATIONS on spontaneous benign mammary growths in intact RIIIb breeding females lacking milk-factor suggested that their prevalence increased with parity rather than with age. Up to 5 litters the effect of age appeared to be strong. Only 1 out of 24 females was affected at 11 months and 12 out of 24 at 20 months but this impression was not supported by counts after 6 or more litters. When 21 breeders had borne at least 6 litters the number affected at 11 and at 20 months of age was 10 and 9 respectively (Pullinger, 1955). It was probable that any effect of age on incidence was due to a continuation of oestrus cycles when breeding was stopped after the smaller number of litters. To test this conclusion mice that had borne various numbers of litters were ovariectomised at 9 to 10 months before viable growths were expected to appear. One group had no further treatment ; oestrone was applied to another. All were killed at 20 months and the mammae examined. It was found that ageing alone of the ovariectomised mice resulted in a fifteen-fold reduction in incidence of viable benign growths. No malignant tumours appeared after ovariectomy alone but the number of breeders used was too small to judge whether carcinoma had been suppressed. When oestrone in doses of 0·1 to 0·15 mg. was substituted after spaying, incidence, total number and variety of growths, both benign and malignant, was that to be expected in intact multiparae segregated at the same age.

### METHODS.

The mouse strain, breeding, examination of mammae and ovariectomy were the same as previously described (Pullinger, 1947, 1949*a*). Only pure line progeny were used. Breeders were segregated from males 1 month before spaying. Age groups used were 9 to 10 and 11 months old breeders. The last was needed to find out if benign growths could be prevented by ovariectomy at 11 months and if not, whether they would survive for the remainder of the experimental period. When oestrone was substituted it was applied in a 0·01 per cent solution in acetone to the skin of the back from which hair had been clipped. Doses of 0·05 ml. were applied once weekly for 20 to 30 weeks, the actual total dose varying from 0·1 to 0·15 mg. Four months were allowed to elapse before the mammae were examined to ensure regression of any physiological response.

### RESULTS.

Before stating results it can be confirmed that no restoration of oestrus cycles, nor adrenal hyperplasia or tumours have ever been seen in mice of this strain after ovariectomy at puberty. None has been spayed in the first week after birth. The results of 2 groups, 3 to 5 litters and of 6 or more litters by 9 months of age correspond with those in intact mice in Table I of the previous paper (Pullinger, 1955). The mammae of 16 ovariectomised breeders of 3 to 5 litters were free of benign growths of any kind when examined at 20 months. A mammary gland of 1 out of 17 mothers of at least 6 litters contained 1 fibrous adenoma. In 45 intact breeders 21 were affected with benign growths ; in the two ovariectomised groups together one breeder out of 33 was affected, a fifteen-fold reduction in incidence after ovariectomy.

When oestrone was applied for 6 months after ovariectomy in total doses of 0·1 to 0·15 mg. the mammae of 14 out of 31 breeders of 3 to 8 litters, examined at 20 months, contained benign growths. Of these 11 were purely acinar, 6 were adenoacanthomas. At 18 months one of these treated breeders developed a carcinoma—a malignant adenoacanthoma. This female was derived from a first litter and had herself borne 4 litters. The incidence of benign growths was restored by oestrone to about the number and variety to be expected in intact breeders. The single carcinoma did not represent any increase above the usual incidence.

When ovariectomy was delayed until 11 months and the mammae examined at 20, 6 out of 13 breeders of 6 or more litters were affected with benign growths, 2 of which were viable adenoacanthomas and 4 were foci of purely squamous proliferation. These observations suggest that 11 months is critical for development of viable benign growths, that is to say some at least cannot be suppressed when ovariectomy has been delayed to that age. They prove also that, having formed, viable growths can persist without ovarian hormones for 9 months. Previously the survival time after ovariectomy had not been tested for longer than 4 months.

In some earlier experiments which were prematurely ended by an epidemic of Tyzzer's disease, 8 breeders had been ovariectomised immediately after their fourth litters at 4 to 6 months of age. After intervals which varied from 6 to 11 months, these 8 were treated with the same dosage of oestrone. Three developed purely acinar growths found at 20 months. These few observations support the view that benign viable growths which appeared after ovariectomy and oestrone treatment were not preformed and thereafter merely maintained. All the substitution experiments suggest that potentially benign, neoplastic foci, previously determined by multiple pregnancies, were promoted or developed by oestrogen. This action is analogous to promotion in experimental carcinogenesis of skin warts (Friedewald and Rous, 1944).

In other unpublished experiments a dose of oestrone 10 times larger than that used here failed to induce mammary carcinoma or to cause malignant change in benign growths in 37 breeders which had been ovariectomised at 16 months, treated to 20 and allowed to survive. This dose was sufficient to provoke lobular-alveolar development and milk-like secretion (Pullinger, 1949*b*).

### COMMENT.

In comparing the effects of age and of oestrogen substituted after ovariectomy the inactive squamous epithelioses have been ignored because it is probable that they are dead structures which have no further significance. They are, however, indicators of the earliest tendency to pathological proliferation.

At one time, during the course of these observations, carcinoma had appeared only in females derived from first litters. An association was then sought between liability to carcinoma and the possible influence of the X-zone of the adrenal overlapping in time the early development of the mammary gland. Extracts of X-zone from young mice were injected daily for 3 days into pregnant multiparae from the fourteenth to sixteenth days of pregnancy judged by the vaginal plug test. One only out of 18 female progeny developed a mammary carcinoma after unrestricted breeding. Later, carcinoma was found in breeders from litters other than the first and an association could no longer be suspected.

Owing to scarcity of malignant mammary tumours in the RIIIb strain, and thus the need for greater numbers of animals, the methods here used to relate tumour incidence and hormone action could not be applied.

SUMMARY.

1. The incidence of benign mammary growths in old female mice was decreased fifteen-fold by ovariectomy after breeding. Malignant growths were too rare to apply the same methods to test the effect of age after ovariectomy on their incidence.

2. When oestrone was substituted after ovariectomy the proportion of benign and of the only malignant mammary tumour which occurred was comparable with that in intact breeders in old age. Benign were not converted into malignant growths by oestrone in the doses used.

The work was done while holding the Alice Memorial Fellowship and with a grant from the Imperial Cancer Research Fund.

REFERENCES.

FRIEDEWALD, W. F. AND ROUS, P.—(1944) *J. exp. Med.*, **80**, 101.

PULLINGER, B. D.—(1947) *Brit. J. Cancer*, **1**, 177.—(1949*a*) *Ibid.*, **3**, 494.—(1949*b*) *Lancet*, ii, 823.—(1955) *Brit. J. Cancer*, **9**, 613.